# On Some of the Injuries and Diseases of Joints, as Illustrated by Cases from Guy’s Hospital

**Published:** 1858-12

**Authors:** Thomas Bryant

**Affiliations:** Assistant-surgeon, etc., to Guy’s Hospital


*On some of the Injuries and Diseases of Joints, as Illustrated by cases from Guy's Hospital.* By Thomas Bryant, F. R. C. S., Eng. Assistant-surgeon, etc., to Guy's Hospital.

If the experience of the Metropolitan Hospitals could be carefully collected, analyzed, and compiled, for the short period of five years, the amount of information and benefit which would be given to the world, would, without doubt, be of immense value. And although at this time I am not disposed to enter into the question of how far the public have a right to demand for the record of that experience, there is no doubt that men who stand in a position favourable far observation, are in some degree debtors to the professional public, and should give them when able, the benefit of their experience.

Having now for upwards of five years held the post of Surgical Registrar to Guy's Hospital, and thus been enabled to observe and take notes of every Surgical case admitted during that perod within its walls, it is my intention to select from my notes of upwards of two thousand cases of injuries and diseases of the joints, such examples of interest as may present themselves, and which will illustate the different points to be alluded to in the following papers. And, as it is my hope, that cases will be found adapted to illustrate most of the injuries and diseases of joints, I shall commence the series by the subject of Wounds of Joints.

### WOUNDS OF JOINTS.

The experience and knowledge of wounds of joints which the civil Surgeon acquires must necessarily be somewhat limited, and it is to his military brethren that he generally turns for the information he may desire, as to the symptoms, results, and treatment of such injuries.—My notes of cases admitted into Guy's Hospital, however, afford instances of all the different wounds to which joints are exposed, and include the incised, lacerated, and punctured, together with examples of compound dislocation, compound fracture with laceration into the joints, and gunshot wounds.

The symptons which generally follow a wounded joint are very variable, and are by no means in their severity proportionate to the extent of injury: a wound may in one subject be followed by but slight inflammatory symptoms, and in others acute suppuration of the joints with its attendent dangers may be the sequence. In one case, even of severity, the joint may perfectly recover, in another its loss by disorganisation or by anchylosis may be the end; the constitution and habits of the patient, in these cases as in others, having a marked influence in their result, independent of any treatment, however skillful.

*Case* 1.—The first case I shall relate is one of a severe character as regards the wound, but of simplicity with respect to symptoms: the joint opened was the knee, but its result was all that could be wished. A healthy, but strumous-looking girl, aged 9 years, when running fell upon an oyster-shell, which produced a wound one and a half inches long, above and to the side of the patella, exposing the joint, as indicated by the flow of its synovia. The knee was kept at rest, and fixed by sandbags at an obtuse angle, sutures applied to the wound, and a piece of wet lint applied. All went on well, with but slight effusion into the joint. Upon the fourth day a straight splint was applied, and upon the twelfth some extra synovial inflammation took place. Antimonials and salines were given with benefit, and in four weeks all inflammatory symptoms disappeared. Tonics were subsequently administered, and in four months from the admission, she left with a good, but slightly stiff joint.

*Case* 2.—The second case is one of a lacerated wound into the knee-joint, and well illustrates the value of the application of cold in subduing inflammatory action.

A man, aged 27, in a quarrel with his wife, received a blow upon his left knee from a china basin, which she threw at him. An oblique lacerated wound was the result, extending across the joint from above downwards and inwards, laying it completely open.

Sutures and strapping were applied, but only sufficient to keep the parts in apposition, and the joint kept cool by a process of irrigation, or the constant trickling of cold water over the part covered with a layer of lint. A mercurial in the form of the hyd. c. cret. gr. ii., pulv. Doveri, gr. v. was given twice a-day, and perfect rest enforced. For three days all inflammatory action was checked, and the wound healded ; but by accident the application of the cold was omitted for one night, and severe inflammation followed ; this, however, was speedily subdued by the re-application of the remedy; and in one month the man left the Hospital with a sound joint.

*Case* 3.—The third case is one of injury to the elbow, equally fortunate in its termination as the preceding; and affords a good example of the power of nature to produce a cure assisted but slightly by art; rest being the chief element of treatment.

A man, aged 17, fell down a cellar upon some empty bottles; a portion of the glass perforated the left elbow beneath the external condyle, producing a wound an inch long. Some slight hæmorrhage followed, accompanied with great faintness. The wound was brought together by sutures, and water dressing applied, and the arm placed in a semiflexed position. But slight costitutional disturbance followed, and the wound healed rapidly, the joint for the first four days being slightly swollen. After two weeks some slight movement of the joint was permitted, all symptoms of injury having subsided, and after one month's rest in the Hospital he left with the joint as sound as its fellow.

The four next cases are punctured wounds, and, as might be expected, are of a more severe character. The accident in three was followed by acute inflammation of the joints, and in one by inflammation of a less severe character. In one of the former, amputation was resorted to with immediate success, although the patient died of some secondary disease a year afterwards. In the second, a good recovery was made; and in the last death rapidly ensued.

*Case* 4.—A boy, aged 10 years, fell out of a window upon a brass spike, the point perforating the joint close to the patella. Some oily fluid made its escape after the accident. The child was kept perfectly quiet; the joint rapidly enlarged, and some fever was present, but no Medical advice sought for two weeks; when he was brought to the

Hospital as he made no progress. The joint was then painful and swollen but the constitutional disturbance was very slight.—Ice was applied in a bladder, and rest enforced. In three days all swelling had subsided, and in two weeks he left with a healthy joint.

*Case* 5.—A man, aged 36, during a fight was stabbed in the right knee with a fork; the prongs entered quite two inches upon the inner side; no bleeding followed, but the joint became rapidly painful and enlarged. For three days he rested at home, and then sought advice When admitted the joint was enormously swollen, and exquisitely painful; the skin was hot, and pulse full Thirty leeches were applied, and purgatives with antimonials, and colchicum given. After three days the symptoms abated, altuough the pain was still intense, but relieved by opium. The swelling gradually disappeared, and on the 14th day a blister was applied with decided benefit, for upon the 16th the joint had nearly regained its natural size. Upon the 26th the joint was strapped up; and in six weeks from his admission he left with a sound joint.

*Case* 6.—A man, aged 65, of a bad habit, when at work, making chairs punctured his right knee with an adze; acute inflammation and suppuration followed, uninfluenced by the treatment adopted, viz: leeching first, with antimonials and mercury; and subsequently tonics, and support. The powers rapidly gave way, not allowing any operative interference; and after five weeks he died.

*Case* 7.—A man, aged 27, of a strumous aspect, punctured the inner side of his knee with an adze; enlargement of the joint rapidly followed, unattended, however, with very severe pain. Several weeks after the accident he applied to Guy's, with the joint enlarged and evidently suppurating. The joint was opened freely, as the pus was burrowing upwards and downwards: erysipelas unfortunately made its appearance preventing amputation. After a severe struggle he rallied and in about eight months, amputation was performed; from this he rapidly convalesced; about six months after, an abscess appeared in the opposite hip, which burst, and from this he gradually sunk.

From the cases just quoted we may gather a very fair idea of what we may expect after wounds of joints, and form a just one of the principles which should guide us in their treatment.

In healthy and young subjects, as the first five cases illustrate, the inflammation that follows such an injury will vary in its intensity; but will generally, by early and proper treatment, be kept within a dangerous limit.

Page(s) missing

Page numbers 241-244 omitted

In the first four cases the inflammation was never of a very acute character, although in case 2, the omission of the application of cold indicated what it would be if left untreated.

In case 5, also, the symptoms set in which marked severity, and were subdued only by a prompt and energetic treatment.

Case 6 shows the result of such an accident in an old and cachectic man; the joint proved unable to resist the inflammatory action which the injury is prone to induce, and the powers of life unable withstand the drain upon its strength.

Treatment in such cases seems of little service, the rapidity of the diseases preventing their taking effect. In case 7 we have a subject as unfavorable for such an injury as could be imagined; the inflammatory action induced by the puncture was sufficient to set at work the latent disposition to strumous disease to which he seemed naturally liable. Rapid disorganisation of the joint was the result; and although the extra drain upon an exhausted system, as caused by erysipelas, proved a temporary impediment to any operation, still he rallied after the removal of the limb, and recovery might have been expected if the tendency to strumous disease had not manifested itself in other parts. In such a subject any injury would have been sufficient to make manifest his general tendency to disease, and a wounded joint was too serious for his weak powers to overcome.

*Treatment.*—The treatment that should be adopted in these cases is not complicated. Absolute rest is indispensable, and without it all other treatment is of no value. In simple cases where signs of inflammatory action are very slight, as in case 3, but little else is requisite, a cold lotion or wet rag only being necessary. The joint should be placed in the position which is most comfortable, a slightly flexed one being, as a rule to be preferred, and kept there by means of sand-bags or splints. The wound in all cases should be speedily closed, and its edges adapted, if necessary, by sutures. All probing the wound, bandaging and strapping the joint, should be avoided, as being not only unnecessary, but absolutely injurious. The local treatment to be preferred in most cases is cold, not generally in a dry form as ice in a bag, although this is sometimes of value (*vide* case 4); but applied as in case 2, by allowing a gentle and constant trickling of water over the part, covered with a layer of linen.

The comfort of this application is only equalled by its value, and case 2 well illustrates its beneficial influence. Pain ceases, or is considerably diminished immediately after its full action has been experienced and the delight that I have felt at its success is surpassed only by the expressions of the patients.

In some cases, when not seen at an early period, leeching must be freely employed, followed by some warm application, such as a poultice, or which is better, a hot wet flannel.

The constitutional treatment in some cases must be most energetic. In acute cases, opium, antimonials and colchicum, are of great value, mercury having no time for action. In the more chronic form, some mercurial, such as hyd. c. cret. in three of four grain doses for an adult, with an equal quantity of Dover's powder every four hours, may be required. This with purgatives and low diet is all that is necessary.

When the acute symptoms have subsided a blister will occasionally hasten the absorption of the effused fluid, and the application of pressure by strapping will generally effect a cure. In weak subjects, tonics will be called for, and small doses of the iodide of potassium with the syrup of the iodide of iron, in some bitter infusion, is decidedly the best. When suppuration ensues, other treatment must be adopted, the pus should be speedily evacuated, and the question of some other operative interference must soon occur; but the discussion of that question is not now my object.

Support and tonics must then be freely given, and the case assumes a different aspect, and must be classed amongst those of disorganized joints.

Wellington-street, London-bridge.—*London Med. Times & Gazette.*

